# Interactions between Bacteria and *Aspergillus fumigatus* in Airways: From the Mycobiome to Molecular Interactions

**DOI:** 10.3390/jof9090900

**Published:** 2023-09-01

**Authors:** Anne Debourgogne, Lorra Monpierre, Khadeeja Adam Sy, Isabel Valsecchi, Jean-Winoc Decousser, Françoise Botterel

**Affiliations:** 1 UR 7300, Stress Immunité Pathogène, Université de Lorraine, 54000 Vandoeuvre les Nancy, France; anne.debourgogne@univ-lorraine.fr; 2 Unité de Parasitologie-Mycologie, Département de Prévention, Diagnostic et Traitement des Infections, CHU Henri Mondor, Assistance Publique des Hôpitaux de Paris (APHP), 94000 Créteil, France; lorrafelly.monpierre@aphp.fr; 3 UR DYNAMYC 7380, Faculté de Santé, Univ Paris-Est Créteil (UPEC), Ecole Nationale Vétérinaire d’Alfort (ENVA), USC Anses, 94700 Créteil, France; khadeeja.sy@inserm.fr (K.A.S.); isabel.valsecchi@u-pec.fr (I.V.); jean-winoc.decousser@aphp.fr (J.-W.D.); 4 Institut National de la Santé et de la Recherche Médicale (Inserm) U955, 94010 Créteil, France; 5 Department of Infection Control, University Hospital Henri Mondor, Assistance Publique—Hôpitaux de Paris, 94000 Créteil, France

**Keywords:** *Aspergillus fumigatus*, *Pseudomonas aeruginosa*, *Stenotrophomonas maltophilia*, polymicrobial biofilms, mixed biofilm

## Abstract

Interactions between different kingdoms of microorganisms in humans are common but not well described. A recent analysis of the mycobiome has described the presence of different fungi and their positive and/or negative interactions with bacteria and other fungi. In chronic respiratory diseases, these different microorganisms form mixed biofilms to live inside. The interactions between Gram-negative bacteria and filamentous fungi in these biofilms have attracted more attention recently. In this review, we analyse the microbiota of the respiratory tract of healthy individuals and patients with chronic respiratory disease. Additionally, we describe the regulatory mechanisms that rule the mixed biofilms of *Aspergillus fumigatus* and Gram-negative bacteria and the effects of this biofilm on clinical presentations.

## 1. Introduction

A biofilm is a community of more or less complex microorganisms adhered to each other and to a given surface by a characteristically adhesive and protective extracellular matrix they secrete [1]. Depending on the microorganisms involved, biofilms are implicated in both the healthy microbiome and disease states. In this polymicrobial environment, microorganisms have developed complex mechanisms to promote their survival, in particular, to defend themselves against competing organisms [1]. Describing such microbial defensive mechanisms provides a better understanding of their pathogenesis and helps identify which lines of research to follow for the development of antibiofilm therapeutics.

Polymicrobial biofilms and their environment facilitate microbial interactions, cooperative metabolic functions, and cell–cell communications [2]. However, such cooperative or antagonistic microbial interactions can significantly affect microbial virulence, host interaction, and antimicrobial resistance [3,4]. Their growth mode confers phenotypic traits that favour microbial survival in hostile conditions facing antimicrobial drugs and host immunity. For instance, biofilm cells can tolerate antimicrobial and biocide concentrations of 100 to 1000 times higher than what planktonic cells can resist [5]. This tolerance is phenotypic and can be reversible or not, i.e., when biofilm cells disperse and resume a planktonic state (without biofilm) they lose such tolerance [3,6]. Biofilms are also remarkably resistant to host defences and environmental stress conditions, allowing them to persist in different environments [3].

Since the early 1980s, the role of biofilms in microbial pathogenesis has become increasingly evident and the current estimation indicates that up to 60–70% of nosocomial infections are associated with biofilms [3] often formed of bacteria species or even yeasts and bacteria [7,8]. In humans, biofilms build up mostly on epithelial, dental surfaces, or abiotic surfaces such as external devices. Sometimes the microorganisms form aggregates that colonise the lumen of the respiratory tract, for example in chronic respiratory infections such as asthma, chronic obstructive pulmonary disease (COPD), cystic fibrosis (CF), bronchiectasis …) [3].

In chronic respiratory diseases, *Aspergillus fumigatus* remains the most filamentous fungus detected and is almost always accompanied by bacteria, especially Gram-negative bacilli, such as *Pseudomonas aeruginosa* and others.

In this review, we will focus on mycobiota analysis in chronic respiratory diseases and the different mechanisms involved in bacteria–fungi interactions, particularly those between Gram-negative bacilli and *A. fumigatus*.

## 2. Pulmonary Mycobiota and Chronic Respiratory Infections

The lung microbiome, believed to be stable or at least transient in healthy individuals, is now considered a polymicrobial component contributing to dysbiosis and potentially to disease pathogenesis. Advances in culture-independent methods, especially next-generation sequencing, helped reveal the significant impact of lung mycobiome on the clinical presentation of chronic respiratory diseases, such as asthma, COPD, CF, and bronchiectasis [9]. By interacting with the bacteriome and/or virome, the respiratory mycobiome appears to be a cofactor of inflammation and host immune response and therefore, may contribute to lung function impairment and disease progression [9].

The high similarity of the fungal composition of the lower respiratory tract of healthy individuals with that of their dwellings is consistent with the hypothesis that the domestic environment is one of the most significant determinants of the human mycobiome composition [10,11]. The indoor environmental mycobiome is a mix of outdoor fungal components and human (and pet) mycobiome. The human–environment exchange of fungi depends on the specific factors the fungi encounter in each environment (temperature, nutrients, pH, immune response, etc.). Therefore, some fungi are more prevalent in the lungs than in the environment, like *Rhodotorula mucilaginosa*, *Cryptococcus neoformans*, or *Aspergillus* spp., whereas others are more abundant in the air or house dust, such as *Malassezia restricta*, and Capnodiales [9]. The health status of individuals, in particular their inflammatory status, may influence the diversity and richness of the mycobiota rather than its composition [10].

More and more studies are focusing on the composition of microbiota as a new element to consider in chronic respiratory infections and diseases. In asthma, there is strong evidence that the pulmonary and intestinal mycobiomes play a crucial role, highlighting the importance of multi-kingdom microbial interactions in the pathophysiology of asthma [12].

A recent study has shown that a highly variable fungal community dominated by *A. fumigatus* and *Candida albicans*, the two main components of the normal airway mycobiota, colonises the airways. However, notable shifts in the fungal balance within communities and between lung compartments were associated with asthma status (asthmatics, with and without fungal sensitisation, and healthy controls), longer asthma duration, and biomarkers of inflammation. The asthmatics’ airway mycobiota was dominated by a relatively small number of species, mainly *A. fumigatus*, *C. albicans*, and *Mycosphaerella tassiana*, and was distinct from the oropharyngeal mycobiota and air samples [13].

In CF, many bacteria and fungi are implicated in respiratory infections. Two recent studies from the prospective multicentre cohort of French CF patients (MucoFong) showed an interest in studying the mycobiome and bacterial–fungal interactions. The respiratory tract of CF patients is now considered a polymicrobial niche, and advances in next-generation sequencing have made it possible to analyse its microbiota and mycobiota. It has been known for many years that the decline in respiratory function is associated with a decrease in bacterial diversity [14]. More recently, a similar link has been demonstrated with fungi [15]. The inter-kingdom network showed three main clusters organised around *Aspergillus*, *Candida*, and *Scedosporium* genera. *Aspergillus* and *Malassezia* as relevantly associated with CF pulmonary exacerbation, and *Scedosporium* plus *Pseudomonas* with a decline in lung function [14]. Patients with fungal bronchitis in CF exacerbations had the highest fungal biomass dominated by communities of emerging filamentous pathogens, such as *Exophiala dermatitidis* and *Scedosporium* complex, compared with subjects without CF. The clinical subgroup of fungal bronchitis was often characterised by severe symptoms that were difficult to treat [16]. CF prognosis has dramatically improved over the past few decades, hence the urgent need to re-evaluate the prognostic factors for CF progression and to include a systematic assessment of fungal colonisation and/or bacteria-associated infections. For instance, *P. aeruginosa*, *Achromobacter xylosoxidans*, *Stenotrophomonas maltophilia*, and *C. albicans* have been associated with increased disease severity. Such results highlight (i) the potential pathogenicity of species often derived from the oral cavity of CF patients and usually considered clinically insignificant, and (ii) the polymicrobial signature of CF airway colonisation which may contribute as a collective entity to lung function decline [17].

In COPD, the mycobiome has been little explored so far. A distinct mycobiome profile is observed in patients compared with controls, as evidenced by increased α-diversity. Significant airway mycobiome differences, including greater interfungal interaction (by co-occurrence), were detected in patients with very frequent COPD exacerbations (three or more exacerbations per year). The airway mycobiome in COPD is characterised by specific fungal genera, *Aspergillus*, *Curvularia* and *Penicillium*, which were associated with exacerbations and increased mortality [18]. A longitudinal study demonstrated that the pulmonary mycobiome was less stable than the oral mycobiome, and neither COPD diagnosis nor intercurrent antibiotic use seemed to influence stability [19].

All these articles have explored the impact of these diseases on or in association with the lung mycobiome. The authors of these articles describe a reduction in bacterial and fungal diversity during the course of these chronic respiratory diseases, which is proportional to the patient’s respiratory function. For fungi, but this is also true for bacteria, reduced diversity may be caused by an overgrowth of a single fungal species, or by the loss of rare species that comes with a reduction in overall fungal abundance [15,20]. The airway mycobiome in COPD would be most useful in identifying patients most at risk of long-term adverse consequences, including risk of frequent exacerbations and higher mortality [18]. Available data could suggest that specific mycobiome profiles do exist in cases of chronic respiratory disease, but these data should be treated with extreme caution, due to the heterogeneity of the studies. Moreover, no significant changes were seen in airway mycobiome profiles, alpha diversity, or beta diversity, suggesting that treatment for an acute exacerbation did not influence the lung mycobiome [18].

## 3. Limitations of Studies and Laboratory Methodologies

Our review mainly described in vitro interactions between *A. fumigatus* and Gram-negative bacteria. However, the microbiome is a complex entity of different bacterial species, yeasts, and filamentous fungi (and even viruses) determining a new environment on the basis of their mutual interactions combined with many host factors (immune modulators, virulence …). This complexity introduces many biases into the current research.

Lack of Standardisation and Reproducibility: One of the primary concerns in microbiome studies is the lack of standardised methodologies, as described by our team in 2020 about DNA extraction [21]. Each study may use different sampling techniques, especially for sputa, sequencing platforms, and data analysis pipelines, leading to significant variations in results. Multiple gene loci have also been targeted for fungal gene profiling including 18S ribosomal rRNA (small subunit) and 28S rRNA region (large subunit), even if the two internal transcribed spacer units 1 and 2 (ITS1/ITS2) are used most commonly to detect fungi. Similarly, there are variations in micro and mycobiome analysis between individuals and protocols. To date, there is no established gold standard protocol to evaluate the inter-kingdom interactions between bacteria and fungi [22]. Moreover, replicating findings from one study to another has often proved challenging, raising questions about the reliability and reproducibility of microbiome research.

Correlation vs. Causation: Microbiome studies frequently highlight correlations between specific microbial compositions and various health conditions. However, it is crucial to recognise that correlation does not imply causation. Disentangling the cause-and-effect relationship between microbiota and health outcomes remains a formidable task. Many studies lack the rigorous experimental designs necessary to establish causality, leaving room for misinterpretation of the findings. Indeed, an important shortcoming of all studies is their retrospective or cross-sectional design, which is not appropriate to draw any conclusion regarding mycobiome as a causal factor. Despite difficulties in performing such studies, more prospective, multicentric, large, and longitudinal studies over a long time period are required to shed light on the role of the composition of fungal or other microbiota in different chronic respiratory diseases.

Confounding Factors: Human microbiomes are influenced by a myriad of variables, including age, diet, lifestyle, geography, genetics, and medication usage. Untangling the impact of each factor on microbial communities presents a significant challenge. The complexity of these interactions can often lead to spurious associations or confounding effects, obscuring the actual relationships between microbiota and health.

Sample Size and Selection Bias: Numerous microbiome studies suffer from small sample sizes, making it difficult to draw robust conclusions. In this case, the statistical power to detect any differences in the mycobiota between cases and controls is low. Additionally, the process of selecting study participants can introduce bias, which may not be adequately addressed in the analysis. These limitations can affect the generalisability of results and hinder the broader applicability of the findings to diverse populations.

Publication Bias: Like any other field of research, microbiome studies are susceptible to publication bias, where positive or significant results are more likely to be published than negative or non-significant ones. This bias can skew the perception of the overall evidence, leading to an overestimation of the impact of microbiota on human health.

Overemphasis on Specific Microbes: Some studies tend to overemphasise the role of individual microbial species or groups without considering the intricate interactions within the entire microbial community. This reductionist approach may oversimplify the complex dynamics of the microbiome, potentially overlooking the broader ecological significance of the entire ecosystem.

The strength of interactions between microorganisms is therefore difficult to analyse with current bioinformatics tools, especially between bacteria and fungi. Another way to analyse these interactions is to do so in vitro in pairs. The most studied interaction between *A. fumigatus* and bacteria, in the context of chronic respiratory infections, is that between *A. fumigatus* and *P. aeruginosa* in cellular models and/or in vivo models. In this case, interactions were often analysed as mixed biofilm or polymicrobial biofilms.

## 4. Formation of Mixed Filamentous Fungi–Bacteria Biofilm

**Figure 1:**
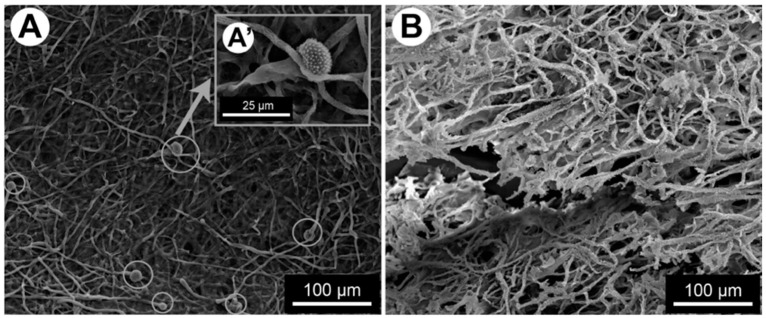
Conidiation and phenotype of *A. fumigatus* in the mixed biofilm visualised on SEM. (**A**) 24-h-old single *A. fumigatus* biofilm (**A’**) zoom on the presence of conidial head (**B**) 24-h-old mixed biofilm of *A. fumigatus* and *S. maltophilia* [25].

A study compared a 24 h *A. fumigatus* biofilm alone or in the presence of *P. aeruginosa* and showed that *A. fumigatus* produced extensive and highly adherent mycelial growth with extensively branched hyphae forming a three-dimensional network of mycelium. In contrast, the monomicrobial biofilm culture of *P. aeruginosa* was weakly adherent. The mixed biofilm of *A. fumigatus* and *P. aeruginosa* showed a hazy background in which many *P. aeruginosa* bacteria were embedded in a network of filaments and mesh-like material [23]. In another study on the same pair of microorganisms, the fungal biomass was significantly reduced by 14.5% (*p* < 0.001) compared with the no-bacteria control [24]. Similarly, the interaction between *S. maltophilia* and *A. fumigatus* gave close biomass data and slowed down the growth of *A. fumigatus* in the mixed biofilm and that occurred during the growth phase of *S. maltophilia* causing a significant fungal biomass reduction of 54% between 12 and 16 h [25]. These differences were observed during the maturation phase of the biofilm after about 12 h and up to 24 h. When the fungus was in contact with the bacteria, the hyphal growth was delayed, and the thickness of the mixed biofilm was significantly less than that of the fungal biofilm alone. The hyphal network was less dense in the mixed biofilm and no *A. fumigatus* conidiation was observed at 24 h. Furthermore, the hyphae were atypical, showing atrophied and highly branched structures with shorter branching at the tips [25] (Figure 1).

**Figure 2:**
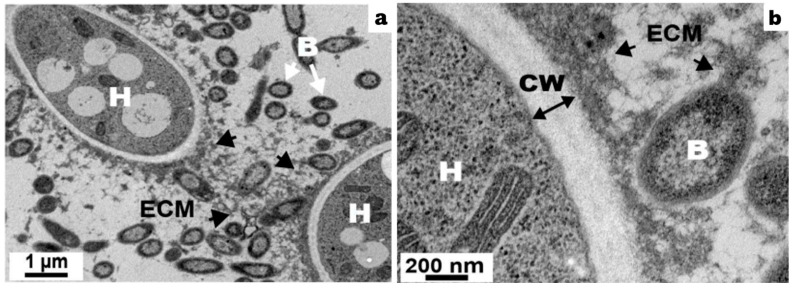
Cell wall thickness of *A. fumigatus* in mixed biofilms. (**a**) scale bar = 1 μm; (**b**) scale bar = 200 nm. Observation on 24 h ± old mixed biofilm by TEM. H = hyphae, B = bacteria, CW = cell wall, ECM = extracellular matrix (indicated by arrow) [25].

Different microscopic approaches (SEM, TEM) showed that *S. maltophilia* cells were in contact with the *A. fumigatus* cell wall and embedded in the extracellular matrix, revealing a direct interaction between the bacterium and the fungus via the extracellular matrix [25] (Figure 2).

In the in vitro mixed biofilm between *A. fumigatus* and *Streptococcus pneumoniae*, the bacteria would disrupt the preformed biofilms of *A. fumigatus* and form a pneumococcal-dominated biofilm. *S. pneumoniae* could suppress both fungal proliferation and mycelium formation in the mixed biofilm without completely killing *A. fumigatus*. When a 48 h preformed fungal biofilm was co-cultured with pneumococci for 24 h, its hyphae were incorporated into the pneumococcal biofilm instead of making the fungal extracellular matrix, and many pneumococci adhered to the hyphae. And when a 24 h preformed fungal biofilm was co-cultured with pneumococci for 48 h, the hyphae were progressively disrupted into fine filaments [26].

Similarly, other filamentous fungi would suffer a negative impact, called antibiosis, if were grown in a mixed biofilm with bacteria. Also, inhibition of fungal growth was also observed in co-cultures of *P. aeruginosa* and *Scedosporium aurantiacum*. Kinetically, the bacteria adhered to the hyphae quite rapidly after co-culture, and within 8 h the bacteria aligned themselves along the length of the fungal hyphae. After 24 h, large clusters of *P. aeruginosa* cells were observed on the hyphal filaments of *S. aurantiacum*. The growth and thickness of hyphae were reduced compared with the control *S. aurantiacum* (i.e., without the bacteria) [27]. In the mixed *Staphylococcus aureus–Fusarium falciform* biofilm, the fungal hyphae and conidia were short and thin, and the number of fungal cells was clearly reduced. As for *S. aureus*, they aggregated in clusters around the fungal structures and some of them were observed on the hyphae, suggesting contact–contact interactions in the mixed biofilm. The biomass of the mixed biofilm was higher than the biomass of the *S. aureus* mono-biofilm (*p* < 0.001), and lower than the biomass of the fungal mono-biofilm (*p* < 0.001) [28]. In another mixed biofilm formed by *A. fumigatus* and *S. aureus*, the electron microscope revealed differences in the texture and distribution of the extracellular matrix (ECM) when compared with single biofilms. In some fragments, the appearance of ECM was porous or condensed or like a film covering the surface of large clusters of cocci [29].

## 5. Extracellular Matrix Production

Biofilms produce their own characteristic extracellular matrix (ECM) that surrounds the biofilm cells and ensures the structural integrity of the microbial community [3]. ECM is usually composed of exopolysaccharides, extracellular DNA, and proteins, although its composition varies considerably depending on growth conditions and microbial species. Exopolysaccharides are the main components of many microbial species that can bind or repel antimicrobial agents through charge and hydrophobicity interactions, thus limiting their intracellular penetration [3].

The exopolysaccharides found in *A. fumigatus* biofilms include galactomannan, galactosaminogalactan (GAG), and α-1,3-glucan [30,31]. GAG is produced by *A. fumigatus* during its vegetative growth to mediate hyphal adhesion to biotic and abiotic surfaces, maintain matrix integrity, and ensure biofilm formation [32,33,34]. Additionally, GAG is a bioactive polymer involved in interactions with host cells. GAG interacts with several immune cells causing masking of pathogen-associated molecular patterns (PAMPs), neutrophil apoptosis, activation of platelet, NK cells, and macrophages [33], and induction of NLRP3-dependent inflammasome in macrophages [34]. In some contexts, GAG is also an anti-inflammatory molecule responsible for IL-1Ra secretion [35,36]. GAG participates in the formation of the extracellular matrix of biofilms, as described by Loussert et al. in a single *A. fumigatus* biofilm of aspergilloma, and in vivo in a mouse model of invasive aspergillosis [30]. Briard et al. documented that GAG was crucial for the interaction between *A. fumigatus* and *P. aeruginosa* since it was responsible for the binding of *P. aeruginosa* to the hyphae and in the biofilm [32]. Overall, GAG is a linear polysaccharide composed of galactose, N-acetylgalactosamine (GalNAc), and its de-N-acetylated form, the galactosamine (GalN). The percentage of each of these components in its sequence is highly variable. Its biosynthesis is carried out by a cluster of five genes (*UGE3*, *SPH3*, *EGD3*, *AGD3*, and *GBT3/GT4C*) [33,34]. The biological properties of GAG, involved in bacteria–fungi interactions or immune cell stimulation, are dependent on its de-N-acetylation. In other words, the percentage of galactosamine (GalN) in GAG would be a key factor in determining *A. fumigatus* virulence [35].

On the other hand, in bacteria, exopolysaccharides are high molecular weight carbohydrate biopolymers, often secreted by cells into the extracellular environment to ensure a variety of functions useful for the bacteria. For instance, *P. aeruginosa* produces three exopolysaccharides: Pel, a cationic polymer of partially deacetylated N-acetylgalactosamine and N-acetylglucosamine (PNAG); Psl, a pentamer of mannose, glucose, and rhamnose; and alginate, an anionic polymer of uronic acids. Pel and Psl play a key role in *P. aeruginosa* biofilm formation by promoting adhesion to surfaces and other cells and by interacting with extracellular DNA and host molecules [29,37,38]. Many *Staphylococcus* species produce deacetylated poly-N-PNAG polysaccharides, which mediate cell-to-cell adhesion and participate in biofilm formation in vitro and in vivo [39]. Deacetylation of hexosamine sugars is required to ensure the adhesive function of GAGs, PNAG, and Pel [40].

## 6. Social Interactions between Gram-Negative Bacteria and *A. fumigatus* in Mixed Biofilm

### 6.1. Antibiosis: The Most Common Social Interaction

There are many social interactions described for bacteria and fungi, such as: (i) physical association that can be planktonic, mixed biofilm, or intrahyphal colonisation and (ii) molecular communications such as antibiosis, signalling and chemotaxis, physiochemical changes, protein secretion, metabolite exchange, metabolite conversion, adhesion, or genetic exchange [1].

Among these social interactions, some are more common, e.g., antibiosis, in mixed biofilms of Gram-negative bacteria and *A. fumigatus*. Antibiosis, as described above, is a biological interaction between two or more organisms brought by the production of a metabolic substance by one of the partners that is prejudicial to the other partner, in terms of growth, morphology or structure.

Antibiosis between different pairs of bacteria and fungi has already been demonstrated and is always detrimental to the bacteria, e.g., *P. aeruginosa* and *Scedosporium/Lomentospora* [36,41], *P. aeruginosa* and *A. fumigatus* [23,40], *S. maltophilia* and *A. fumigatus* [25,42], or *Klebsiella pneumonia* and *Aspergillus* spp. [37]. This inhibitory effect is directly proportional to the number of bacteria [23] or to the distance between the two colonies of microorganisms in the culture [40].

### 6.2. Effect of Antibiosis Induced by the Bacteria on the Development of the Fungi

In the interactions between *P. aeruginosa* and *A. fumigatus*, Manavathu et al. showed that while hyphae were refractory to the fungicidal effect of the bacteria, conidia and spores were sensitive to it and were completely killed.

In contrast, Nogueira et al. showed that *K. pneumoniae* inhibited spore germination and hyphal development of *Aspergillus* species, suggesting that the inhibitory effect of *K. pneumoniae* on *Aspergillus* species is independent of the fungal growth stage. The inhibitory effect was reversible since the fungus resumed its growth upon eliminating the bacteria by antibiotics [37]. A mechanistic hypothesis proposes that in order to withstand the interaction with *K. pneumoniae*, *Aspergillus* adopted a dormant or standby (sleep) mode, during which the genes regulating hyphal development were downregulated, conferring a low energy supply requirement for survival [37].

### 6.3. Strain-Dependent Interaction Effect

Bacteria–fungus interactions can be modulated by factors intrinsic to the isolates present in each association. The antibiosis effect of *P. aeruginosa* or *S. maltophilia* on *A. fumigatus* did not depend on the strain of the fungus [38,42]. However, the inhibition of *A. fumigatus* was related to the source and phenotype of the bacteria isolate. The inhibitory effect of mucoid or non-mucoid CF *P. aeruginosa* isolates was higher than the effect of the non-CF isolates [39,43]. In contrast, small colony variants of *P. aeruginosa* appeared so variable in their ability to inhibit *A. fumigatus* [44]. The antibiosis effect of *S. maltophilia* strains on *A. fumigatus* depended on the bacterial strain and its genotype. *S. maltophilia* strains of genotype 6 isolated from patients, in particular from cystic fibrosis patients, were more virulent on *A. fumigatus* than animal or environmental strains, even at high bacterial concentrations [42].

Many models of bacteria–filamentous fungi interactions have shown that inhibition can also be observed with culture filtrates or supernatants [26,27,28,39,41,43,44,45]. The results suggested that cell–cell contact is not necessary for bacteria to inhibit fungal growth, and those bacterial metabolites and/or extracellular signalling molecules might be involved in this inhibition.

**Figure 3:**
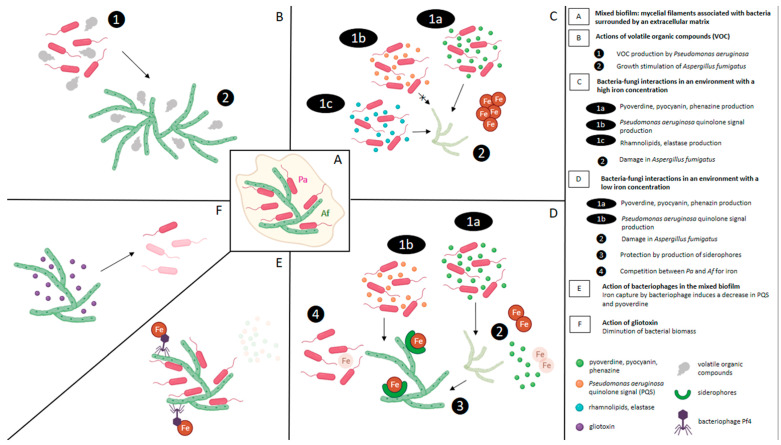
Synthesis of different mechanisms involved in bacteria–fungi interactions, particularly those between Gram-negative bacilli and *Aspergillus fumigatus*.

The different mechanisms described in these bacteria—*A. fumigatus* interactions are developed in the following paragraphs and summarised in Figure 3.

## 7. Effect of Extracellular Soluble Molecules of Bacteria on *A. fumigatus* Development

### 7.1. Role of P. aeruginosa Pyoverdine and Phenazines

Interactions between *A. fumigatus* and *P. aeruginosa* have revealed the antifungal role of the bacteria and the interference with fungal metabolism or growth via molecules the bacteria secrete like the major siderophores, pyoverdine, and phenazines, such as pyocyanin (5-N-methyl-1-hydroxyphenazine), 1-hydroxyphenazine, phenazine-1-carboxamide, phenazine-1-carboxylic acid, and di-rhamnolipids [46]. Upon using pyoverdine-defective mutants, it was possible to prove the key inhibitory role of this siderophore. Studies exploring pure pyoverdine effects corroborated these findings, including the restoration of inhibition by the pyoverdine deletion mutants. A correlation was also observed between the concentration of the produced pyoverdine and the resulting antifungal activity in clinical isolates of *P. aeruginosa* taken from the lungs of CF patients [44,45,47,48]. Pyocyanin exerted a similar inhibitory effect on *A. fumigatus* but not on *S. aurantiacum* [27,45]. A synergistic antifungal activity was described by adding voriconazole combined with *P. aeruginosa* supernatants. The inhibitory effect was mediated by pyoverdine and pyochelin under low iron conditions and pyocyanin under high iron conditions [46]. The key inhibitory mechanism of pyoverdine relied on iron chelation and iron denial to *A. fumigatus* [48].

### 7.2. Role of P. aeruginosa Quinolone Signal (PQS)

PQS 3,4-dihydroxy-alkyl-quinolone is a quorum-sensing molecule of *P. aeruginosa* that plays a coordinating role in many bacterial functions. It is involved in inter-microbial interactions, iron chelation (specifically Fe^3+^), and Fe delivery to the cell membrane in conjunction with siderophores. Under low Fe conditions, PQS inhibited the formation of *A. fumigatus* biofilms. Inversely, in an iron-rich environment, both biofilm formation and *A. fumigatus* growth significantly increased as compared with an iron-poor environment. This phenotype was not observed with an *Aspergillus* mutant not expressing siderophores. PQS production by *P. aeruginosa* could have favourable consequences for *A. fumigatus* in CF patients’ airways where Fe homeostasis is impaired, thus aggravating the disease [49].

### 7.3. Role of Rhamnolipids and Elastase

Elastase and rhamnolipids are major antifungal molecules of *P. aeruginosa*. Both act by increasing cell wall thickness and inhibiting fungal growth. They are controlled by the quorum sensing system and could help *P. aeruginosa* in iron acquisition [50]. Elastase is a metalloprotease that hydrolyses extracellular matrix proteins and degrades components of the host immune response, including lysozyme and proteins involved in bacterial opsonisation. The presence of a fungus stimulates the production of this important virulence factor by *P. aeruginosa* [40].

### 7.4. Role of Volatile Organic Compounds

Communication between microbial species involves not only the release of water-soluble compounds but also the release and detection of volatile organic compounds. Briard et al. were able to demonstrate an unexpected stimulatory effect of *P. aeruginosa* on the growth of *A. fumigatus*. The effect was mediated by dimethyl sulphide, which is a volatile gaseous compound [51,52]. The same observation was documented in the *Scedosporium* species [41].

### 7.5. Role of Bacteriophages

The filamentous bacteriophage Pf, of genus *Inovirus*, is abundantly produced by *P. aeruginosa* biofilm in clinical CF isolates. Bacteriophage Pf4 inhibits the metabolic activity of *A. fumigatus* biofilms. This phage-mediated inhibition would be dose-dependent, reversed by phage denaturation, and would be more pronounced against pre-formed *A. fumigatus* biofilms than against forming biofilms. However, planktonic conidial growth is unaffected. Pf4 can attach itself to *A. fumigatus* hyphae avidly and for a prolonged period of time. Given that Pf4 usually binds to iron, it denies *A. fumigatus* a crucial resource. This inhibition is reversible and could be overcome with supplemental ferric iron [53]. Infection of *P. aeruginosa* by Pf bacteriophage significantly reduced the production of quorum-regulated or unregulated antimicrobial molecules and pyoverdine [54].

## 8. Effect of Extracellular Soluble Molecules of Fungi on Bacterial Development (Figure 3)

### 8.1. Role of Gliotoxin

*A. fumigatus* and *P. aeruginosa* have mutual antagonistic effects. *A. fumigatus* supernatants showed strong anti-*Pseudomonas* activity in which gliotoxin is the major active agent, a fact supported by the inability of a gliotoxin-mutant strain of *A. fumigatus* to inhibit *P. aeruginosa* biofilm formation. Moreover, gliotoxin reduces bacterial biomass. Based on these facts, a broad-spectrum antibiofilm activity was described against other bacteria commonly found in CF airways [55].

### 8.2. Role of A. fumigatus Siderophores

*Aspergillus* retaliates by releasing hydroxamate siderophores that play a central role in the competition for iron between *A. fumigatus* and *P. aeruginosa* [48,56]. Sass et al. studied a supernatant of *A. fumigatus* mutant lacking *sidA* and saw that fungal siderophores were able to protect *A. fumigatus* biofilms from *P. aeruginosa*’s antifungal activity [56]. A lack of iron was the signal to increase the production of fungal siderophores, molecules that specifically chelate ferric iron with high affinity.

## 9. Role of Iron in Biofilm Interactions

Iron is an essential nutrient for both pathogens; hence, the competition between them to acquire it. In pathological situations such as CF, lung mucus acts as an iron chelator, creating an iron-limiting environment for pathogens living in the lungs. However, there are situations where iron availability is not limited, such as in microhaemorrhages and haemoptysis. Under iron-limiting conditions, *P. aeruginosa* released pyoverdine to deny iron to the fungus, thus interfering with fungal biofilm metabolism. Under non-iron limiting conditions, where pyoverdine was no longer produced, *P. aeruginosa* were still able to affect *A. fumigatus* biofilms by releasing pyocyanin toxin. Thus, the bacteria can use different molecules depending on the encountered inter-microbial competition [50].

There is a paradoxical microbial interaction between *P. aeruginosa* and *A. fumigatus* ruled by the amount of iron present in the medium. In an iron-poor environment, *A. fumigatus* biofilm formation was inhibited, whereas, in an iron-rich environment, both fungal biofilm formation and *A. fumigatus* growth were boosted [50].

## 10. Other Mechanisms

### 10.1. Apoptosis and ROS Production

After 24 h of *P. aeruginosa*–*A. fumigatus* interaction, apoptotic effects characterised by elevated intracellular ROS levels, increased depolarisation of mitochondrial potential, and DNA fragmentation mediated by metacaspase activation were observed [43]. The release of phenazines and gliotoxin was associated with ROS production. By inducing differential levels of oxidative stress controlled by metabolites’ redox properties and their environment-dependent activities, phenazines and gliotoxin play a dual role; as toxins with antifungal activities (high levels) and as a conidiation signal in *Aspergillus* development (moderate levels), which was fine-tuned by NapA oxidative stress response pathway [57].

### 10.2. Role of Oxygen and pH Gradients

Biofilm formation establishes an environment with stable gradients of oxygen, nutrients, and pH that provide different localised habitats on a small scale [58]. Such gradients are probably important in chronic respiratory diseases, e.g., CF, where the apical environment of epithelial cells is hypoxic and rather anaerobic. In this setting, *P. aeruginosa* growth is reduced by the anaerobic conditions, and bacterial filtrates are less effective against hypoxic *A. fumigatus*. Without the mixed biofilm, *P. aeruginosa* would therefore be less active in such environments, as would be the case in CF [59].

## 11. Susceptibility of Polymicrobial Biofilms

The polymicrobial biofilm of *P. aeruginosa* and *A. fumigatus* showed differential susceptibility to antimicrobial drugs, whereas this susceptibility did not change in the *A. fumigatus* monomicrobial biofilm. For instance, tobramycin alone and in combination with posaconazole were highly effective against *P. aeruginosa* monomicrobial and polymicrobial biofilms, respectively, whereas cefepime alone and in combination with posaconazole showed excellent activity against *P. aeruginosa* monomicrobial biofilms, and less activity against polymicrobial biofilms, respectively. *A. fumigatus* polymicrobial and monomicrobial biofilms showed similar susceptibility to posaconazole with and without the antibacterial drug [23]. In another study on microbial interactions of *A. fumigatus* and *S. maltophilia*, Roisin et al. demonstrated that polymicrobial biofilms could modulate the antimicrobial response of pathogens. *S. maltophilia* could increase the antifungal susceptibility of *A. fumigatus* to amphotericin B, whereas *A. fumigatus* could protect *S. maltophilia* from levofloxacin [60].

Biofilm growth generates microenvironments that host physiologically heterogeneous cell populations of different growth and metabolic states, or cells subjected to different nutritional or oxidative stresses [61,62]. Metabolically quiescent cells or those expressing adaptive stress responses are less susceptible to antimicrobial killing. Finally, recent studies have reported that Gram-negative bacteria and fungi overexpress antimicrobial efflux pumps. The relative contribution of these mechanisms to biofilm antimicrobial tolerance varies between individual organisms and antimicrobials [3]. For example, the inhibitory effect of *S. maltophilia* on *A. fumigatus* growth was more pronounced in CF strains than in reference strains [60].

## 12. Polymicrobial Biofilms Evasion of Immune System

Microorganisms growing in biofilms readily evade the host immune system through different mechanisms. In vitro studies suggested that microorganisms in biofilms are less recognised by the immune system [63,64,65] and thus resistant to phagocytosis and killing by neutrophils [66,67]. For example, the *P. aeruginosa* exopolysaccharide Psl reduces opsonophagocytosis by inhibiting complement molecules deposition at the surface of the bacteria and promotes intracellular bacterial survival, whereas alginate protects against phagocyte activation, uptake, and killing [68,69]. Similarly, exopolysaccharide GAGs mask b-glucans from recognition by Dectin-1, a receptor of innate immunity, and protect hyphae from neutrophil extracellular traps [35,70].

## 13. Conclusions

Microbes are sometimes colloquially described as “friends”, and even “frenemies” (“Frenemies are enemies who act like friends”), as mentioned by Justine Dees in the ASM editorial brief of 1 February 2019. The word “frenemy” is particularly suited to relationships that defy the limits of commensalism, mutualism, and parasitism. Frenemies perfectly describe some of the relationships between microbes that coexist in an infection, such as bacteria and filamentous fungi. These relationships emerge in mixed biofilms and are mediated by complex regulatory mechanisms controlled especially by iron and other metals. In chronic lung infections, this balance between filamentous fungi may partly explain some fungal complications. While microbiome studies offer exciting prospects for medical and scientific advancements, they are not immune to challenges and limitations. As the field continues to evolve, researchers must address these issues head-on and adhere to rigorous scientific practices to ensure the validity and reliability of their findings. Collaboration among researchers, standardisation of methodologies, and a critical mindset are paramount to realising the full potential of microbiome research and its practical applications in the future.

## Data Availability

Data sharing not applicable.
